# Current strategies for improving osseointegration of mesoporous silica drug delivery systems: A scoping review protocol

**DOI:** 10.1371/journal.pone.0338462

**Published:** 2026-02-04

**Authors:** Adrianna Skwira-Rucińska, Jakub Ruszkowski, Adrian Szewczyk, Magdalena Prokopowicz

**Affiliations:** 1 Laboratory of Molecular Enzymology and Oncology, Intercollegiate Faculty of Biotechnology of University of Gdańsk and Medical University of Gdańsk, Gdańsk, Poland; 2 Department of Nephrology, Transplantology, and Internal Medicine, Faculty of Medicine, Medical University of Gdańsk, Gdańsk, Poland; 3 Department of Physical Chemistry, Faculty of Pharmacy, Medical University of Gdańsk, Gdańsk, Poland; Advanced Materials Technology Research Institute, National Research Centre, EGYPT

## Abstract

Mesoporous silica (MS) is widely recognized as a local drug delivery system in bone-related diseases. Although MS enables controlled or sustained release and improved bioavailability of therapeutic agents, its limited native osseointegration capacity remains a critical barrier to effective bone regeneration. Numerous engineering strategies have therefore been proposed to enhance its biological performance. This scoping review protocol aims to collect studies, published from January 2015 onwards, that report evidence on the osseointegration potential (i.e., osteoconductive, osteoinductive, or proangiogenic properties) of MS bone drug delivery systems. Studies indexed in PubMed, Scopus, and Embase will be screened to identify the strategies used to improve MS-mediated bone regeneration, including structural modifications, formulation into composites, and incorporation of bioactive molecules. A structured analytical framework will be applied to explore how specific design approaches relate to biological outcomes across experimental models *in vitro*, *in vivo*, or *ex vivo*. The resulting scoping review will identify trends and knowledge gaps in strategies intended to enhance the osseointegration of MS bone drug delivery systems, supporting their future development and rational optimization for bone repair.

## Introduction

Mesoporous silica (MS) is an amorphous and porous silica material with pore diameters in the mesoscale, ranging from 2 to 50 nm, according to IUPAC [1]. It is typically synthesized through sol-gel methods and is characterized by its biocompatibility, large specific surface area (up to 1500 m^2^/g), and the presence of surface silanol groups (≡Si–OH), which serve as adsorption sites for the binding of drugs [2]. MS can be classified into ordered and non-ordered types. Ordered MS features a uniform pore arrangement and a narrow pore size distribution due to templating during synthesis [3], whereas non-ordered MS has heterogeneous pores with wide size distribution in the absence of a template [4].

MS has been extensively investigated in local drug delivery to bone tissue [5]. Local delivery of a drug directly to the affected tissue enhances its bioavailability, enables controlled and prolonged release, which in turn improves therapeutic efficacy and reduces side effects compared to systemic administration. Among hexagonally ordered MS, MCM-41 and SBA-15 are the most studied and have been used to local deliver of antibiotics [6–9], bisphosphonates [10], and anticancer agents [11,12], as well as peptides [13] and growth factors [14]. However, despite its drug delivery capabilities, MS exhibits limited potential for bone regeneration.

Osseointegration, the dynamic process of implant–bone integration, serves as the foundation for bone tissue regeneration [15]. Effective bone regeneration requires biomaterials with specific properties. Osteoconductivity refers to the ability to create a favorable microenvironment and scaffold for hosting bone cells [16] while osteoinductivity refers to the capacity to stimulate undifferentiated progenitor cells to differentiate into osteogenic cells [17]. It is well established that osteogenesis (the formation of new bone) is closely coupled with angiogenesis, and these processes must remain tightly coordinated for proper bone function [18,19]. The formation of new blood vessels is particularly critical for healing critical-size defects (> 1 cm) [20]. Therefore, in addition to osteoconductivity and osteoinductivity, biomaterials intended for bone implantation should also exhibit proangiogenic activity to support vascularization and effective bone regeneration.

The lack of sufficient osseointegration potential, and consequently, the limited capacity to regenerate bone tissue, is a common feature shared by all types of MS. Direct implantation of MS into a bone defect often fails to achieve the sufficient regenerative effect, as it does not fully replicate the natural bone microenvironment. Unlike native bone, MS lacks a complex organic matrix, which reduces its ability to support bone regeneration [21]. Another key limitation is its variable degradation rate: if degradation occurs too rapidly, structural support is lost before new bone can form, whereas excessively slow degradation (common for MS) hinders bone ingrowth and delays healing [22]. Moreover, MS lacks bioactive factors needed to stimulate cellular responses [23]. In its powder form, it is dusty and lacks structural integrity to serve as a three-dimensional scaffold for bone cells. Limited proangiogenic activity further restricts vascularization, impairing nutrient and oxygen supply to the regenerating bone and reducing the overall effectiveness of the material [24]. These factors collectively result in poor osteogenesis and osseointegration.

Taken together, MS is an excellent drug carrier; however, it does not inherently exhibit optimal properties for bone tissue regeneration. Therefore, extensive research has been conducted for many years to modify these materials to enhance their regenerative potential [25–30]. However, based on our knowledge and preliminary MEDLINE search, there are no existing systematic or scoping reviews on this topic. While reviews discuss utility of MS as a drug carrier for local delivery, none comprehensively map strategies to enhance the osseointegration of MS-based bone drug delivery systems.

Herein, we provide the protocol for a scoping review that will include publications from the last ten years reporting the osseointegration potential of proposed MS bone drug delivery systems. We will review the influence of various modifications of such systems on their osteoconductive, osteoinductive, and proangiogenic properties. Ultimately, the resulting scoping review will provide up-to-date insights into trends in engineering multifunctional, drug-loaded MS bone implants and will highlight the most promising strategies for enhancing regenerative outcome. It will also examine the experimental models and analytical methods used to evaluate bioactivity, addressing a current gap in comprehensive reviews on modifications of MS drug delivery systems for bone regeneration.

## Materials and methods

To improve the searchability of the proposed protocol, it was preregistered on the OSF platform: https://osf.io/za6u7.

### Review questions

**Fig 1:**
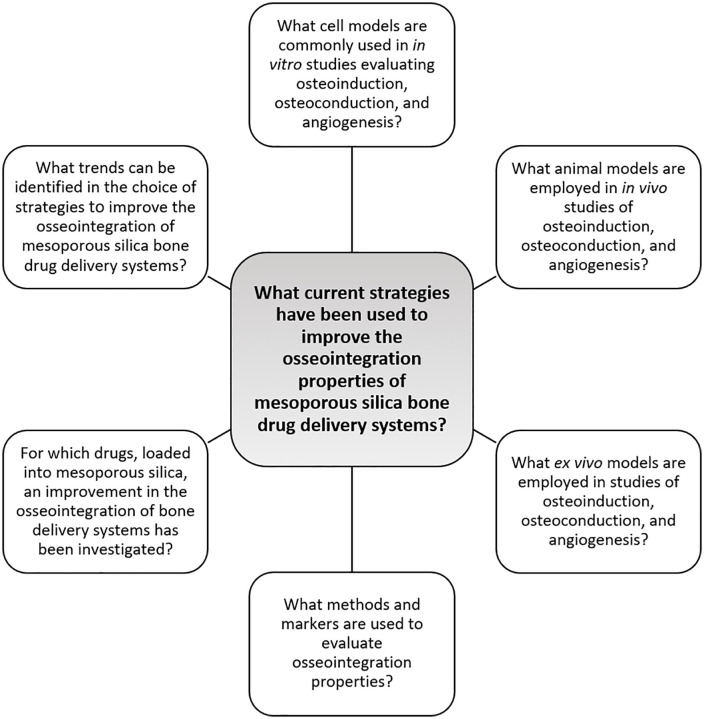
The primary question (center) and secondary questions (surrounding) guiding the scoping review.

The review questions were presented in Fig 1.

### Eligibility criteria

**Fig 2:**
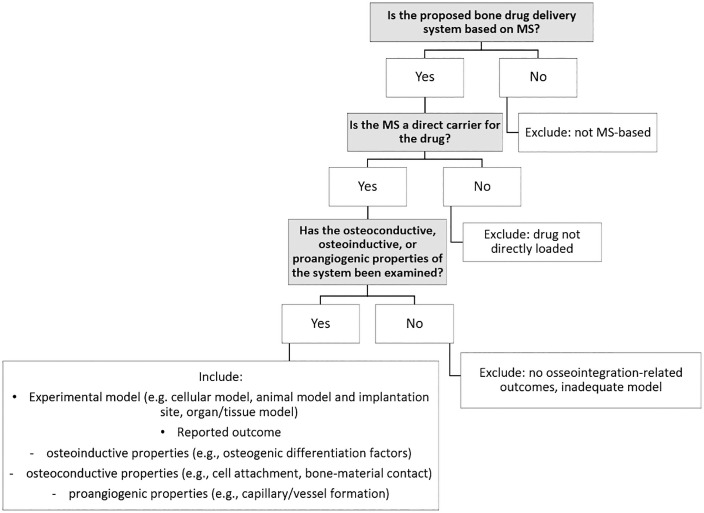
Flow diagram with questions related to eligibility criteria and principal reasons for exclusion.

The criteria are summarized in Fig 2 and described in the following paragraphs.

It is required that the mesoporous silica serves as a direct carrier of the drug in the given system.

### Experimental models

This review includes studies utilizing both *in vitro*, *in vivo,* and *ex vivo* experimental models for osseointegration assessment. *In vitro* studies are included if they utilize primary cells (such as primary human osteoblasts HOB, primary human endothelial cells, e.g., HUVEC and stem cells (including mesenchymal stem cells and endothelial progenitor cells)), or established cell lines (such as human osteoblast hFOB 1.19, pre-osteoblast MC3T3-E1, osteoblast-like MG-63, SaOS-2, U2OS, and endothelial HMEC-1). For *in vivo* and *ex vivo* evaluation, studies using any animal and tissue/organ models to assess bone regeneration or vascularization are considered.

We are interested in biological, cell-dependent studies, as osteoconduction, osteoinduction, and angiogenesis processes depend on cellular activity. If a publication presents only a study of apatite-forming ability in simulated body fluid or other acellular media, it will be rejected, as it will not provide direct evidence of the expected properties of the materials. When it comes to *in vivo* assays all animal models and implantation sites will be considered, both direct into the bone tissue and ectopic sites. *Ex vivo* models include any tissue/organ model that is maintained and studied outside the organism.

### Intervention

This review will consider studies that examine bone drug delivery systems, based on synthetic mesoporous silica, with reported osteoconductive, osteoinductive, and proangiogenic properties. Engineering strategies may include, but are not limited to:

- Structural or physicochemical alterations affecting material properties
- Doping with ions
- Formation of composites with other biomaterials (synthetic or natural)
- Combining with bioactive molecules

Herein, the term “drug” is not limited to well-established definitions proposed by Food and Drug Administration, but it is defined as any active substance, irrespective of whether it is of synthetic or biological origin, that is directly loaded into the mesoporous silica material, thereby confirming its role as a drug carrier. Studies where the drug is not directly carried by the mesoporous silica will be excluded. Similarly, studies examining non-mesoporous silica materials or bioactive glasses will not be considered.

### Control

The review aims to map the effects of MS bone drug delivery systems engineering strategies on osseointegration potential. The controls reported in the publications may vary in type and quality; they will be described to illustrate how the effectiveness of material modifications was assessed.

Typical controls may include, for example:

- Unmodified mesoporous silica
- Commercial bone substitutes
- Standard culture conditions (for *in vitro* studies)
- Empty defects (for *in vivo* studies)
- Native tissue without exposure to the material (for *ex vivo* studies)

The presence or absence of appropriate controls will not be used as an inclusion/exclusion criterion; the primary aim of this review is to map the literature rather than assess the effectiveness of interventions.

### Concept and outcomes

This review focuses on mesoporous silica bone drug delivery systems that follow the undermentioned concepts:

1. Mesoporous silica serves as the direct carrier of the drug
2. Mesoporous silica bone drug delivery system was engineered to improve its osseointegration potential
3. One of the key osseointegration-related outcomes has been examined for them in an *in vitro* cell-based assay, *in vivo*, and *ex vivo* study [3]:
  - Osteoinductive properties (e.g., ability to stimulate undifferentiated mesenchymal stem cells to differentiate into osteoprogenitor cells)
  - Osteoconductive properties (e.g., ability to support ingrowth of sprouting capillaries, perivascular tissue, and osteoprogenitor cells)
  - Proangiogenic properties (e.g., ability to promote blood vessel formation)

Studies must report at least one of these outcomes, evaluated through *in vitro*, *in vivo*, or *ex vivo* experiments. The outcomes may be either primary or secondary endpoints of the studies.

### Context

This review will consider studies examining mesoporous silica bone drug delivery systems for bone tissue applications, including but not limited to bone defect treatment, bone infection therapy, and bone tumor treatment. No geographical restrictions will be applied. As we anticipate that the majority of relevant publications in this field are published in English, we have decided to include only English literature to maintain consistency in our screening and data extraction processes. Such an approach may cause a language bias that risk of which will be acknowledged in the final review.

### Types of sources

This scoping review will consider primary research papers reporting experimental studies. Systematic reviews will be collected to identify relevant references and to map the existence of systematic evidence synthesis in the field, but will not be included in the final data synthesis.

Conference abstracts, narrative reviews, editorials, opinion papers, and articles not reporting original experimental data will be excluded.

### Methods

The proposed scoping review will be conducted in accordance with the JBI methodology for scoping reviews and will follow the PRISMA-ScR reporting recommendations [31,32].

### Search strategy

The search strategy has been designed to locate both published and unpublished studies. Initially, a limited search of PubMed, Scopus, and Embase was undertaken to identify articles on the topic. A complete search strategy for PubMed was developed based on the text words contained in the titles, abstracts, and keywords of relevant articles, as well as synonyms found during manual searches, deducted during discussions between authors, and found in the MeSH and Emtree thesauri. The developed high-sensitive search strategy contains 3 parts:

(1) High-sensitive search of source related to silicon dioxide-based materials – it contains directly stated mesoporous silica materials [both descriptive terms (e.g., “mesoporous silica”, “mesoporous SiO_2_”), names of commonly used materials (e.g., “SBA-15”, “MCM-41”), and brand names of materials of interest (e.g., “AEROPERL 300”, “Syloid XDP 3050”)], terms related to silica-based materials (e.g., “silica scaffold”, “silica xerogel”), and terms related to surface-modified silica materials (e.g., “functionalized silica”, “modified silica”).
(2) Filter for studies related to drug delivery systems (to exclude studies examining silica particles without any drug) – it contains terms such as “drug”, “deliver”, “carrier”, “loaded”, or “release”.
(3) High-sensitive search of sources related to outcomes – it contains synonyms of osteoconduction (e.g., “osteoconductivity”, “osseoconduction”), osteoinduction, and angiogenesis, as well as names of bone cells related to these concepts (e.g., “osteoblast”, “preosteoclast”), bone cell lines used in such studies (e.g., “hFOB”, “MC3T3-E1”), endothelial cell lines used in such studies (e.g., “HUVEC”, “EA.hy926”), proteins related to the concepts of interest (e.g., “osteopontin”, “osteoprotegerin”), genes coding these proteins (e.g., “SPP1”, “TNFRSF11B”), transcription factors related to the concepts of interest (e.g., “RUNX2”, “Sp7”), and methods used to assess outcomes of interest (e.g., “Alizarin Red S”, “von Kossa”)

The source must contain at least one term from each of the three parts to be found by the search strategy.

The search strategy, including all identified keywords and index terms, has been adapted for each included database and/or information source.

The complete search strategy for PubMed has been prepared and is included in S1 Appendix.

Studies published in any language will be included. Since we are interested in up-to-date engineering strategies used to enhance osteoconductive, osteoinductive, and angiogenic properties of MS bone drug delivery systems. The review will include articles published from January 2015 up to the date of the search, which will be conducted immediately following the publication of this protocol.

### Study/Source of evidence selection

Following the search, all identified citations will be collated and initially deduplicated using SR Deduplicator (relax mode). The results will then be imported into Rayyan for a second round of deduplication, followed by screening. Using Rayyan’s screening interface, two independent reviewers will conduct both stages of screening: first titles and abstracts, and subsequently full texts, against the inclusion criteria. Reasons for the exclusion of sources in full text that do not meet the inclusion criteria will be recorded and reported in the scoping review. Any disagreements between the reviewers at each stage of the selection process will be resolved through discussion. If consensus cannot be reached, a third reviewer will provide arbitration. The results of the search and the study inclusion process will be fully reported in full in the final scoping review and presented in a PRISMA flow diagram.

### Data extraction

Each article will undergo data extraction by at least one human reviewer (https://forms.gle/CHYNyndGDCG9fBeE9) and Elicit, an AI-based tool [33]. Any discrepancies found between human- and AI-assisted extractions will be resolved through a second human review. Elicit will be used solely for technical assistance in extracting data from full-text articles. The tool will not perform any interpretation or automated assessment of study outcomes. For reproducibility, the instructions used in Elicit for data extraction will be documented in the supplementary materials of the final manuscript.

The extracted data will include specific details about:

1. Material characteristics:
  - Type of mesoporous silica (e.g., MCM-41, SBA-15, KIT-6).
  - The method of confirming the presence of mesopores in the material (e.g., based on data from another publication or experimental data).
  - Porosity parameters (specific surface area, pore diameter, pore volume, pore arrangement).

Materials in which this has not been confirmed will also be included in the review, but the lack of this information will be noted.

2. Loaded drug.
  - The method confirmed that the drug had been directly loaded into the mesoporous silica material, which served as its carrier (e.g., nitrogen porosimetry (BET, BJH), Fourier-transform infrared spectroscopy (FTIR), X-ray diffraction (XRD, SAXS), thermogravimetric analysis (TGA), nuclear magnetic resonance spectroscopy (NMR, solid-state NMR), Raman spectroscopy).
  - Drug type. In the context of this study, any active substance – regardless of its origin – will be considered a drug when loaded into mesoporous silica. This includes both synthetic compounds (e.g., antimicrobial agents, chemotherapeutics) and biologically derived agents (e.g., proteins, peptides).
  - Indications for the use of a loaded drug in a specific disease or indications for the use of the obtained carrier (e.g., bone infection, osteoporosis, bone tumors, periodontal disease).
3. The strategy used to improve osseointegration of drug-loaded mesoporous silica (e.g., structural alterations, composite formation, incorporation of bioactive molecules).
4. Method used for preparing the final formulation of bone drug delivery system (e.g., sol-gel, co-precipitation, solvent evaporation, melt blending, 3D printing, electrospinning, solvent casting, molecules incorporation)
5. Methods of assessment of the used approach:
  - *In vitro* studies:
    - Cell viability/proliferation assays
    - Migration assays
    - Gene expression analysis (mRNA level)
    - Protein quantification assays
    - Enzymatic assays
    - Angiogenesis assays
    - Microscopic imaging
  - *In vivo* studies:
    - Histological analysis or immunohistochemistry
    - Radiological imaging: X-ray, CT and MRI
    - Biomechanical tests for implant stability (e.g., percussion test, reverse torque)
  - *Ex vivo* studies:
    - Viability/proliferation assays
    - Gene expression analysis (mRNA level)
    - Protein quantification assays
    - Histological or immunohistochemistry
    - Microscopic imaging
    - Mechanical testing of tissue samples (e.g., compression or indentation tests)
    - Biomaterial-tissue interface analysis
6. Experimental models:
  - Cellular models
  - Animal models and implantation sites
  - Organ/Tissue models
7. Reported outcomes:
  - Osteoinductive properties (e.g., osteogenic differentiation factors, bone formation at ectopic sites)
  - Osteoconductive properties (e.g., cell attachment, bone-material contact, bone tissue ingrowth into implantable material)
  - Proangiogenic properties (e.g., capillary/vessel formation, angiogenic factors)

The draft data extraction tool will be modified and revised as necessary during the process of extracting data from each included evidence source. Engineering strategies will be detailed in the scoping review. Since we are interested in the current scope of the literature, authors of papers will not be contacted to request missing or additional data.

### Critical appraisal

Since the critical appraisal, as well as risk of bias assessment, is generally not recommended in the updated methodological guidance for conducting scoping reviews [34], only basic methodological features from included studies will be extracted and reported, specifically: the number of cell lines (for *in vitro* studies), animal species/subspecies together with total number of animals (for *in vivo*/*ex vivo* studies), and the number of replicates/repetitions. Based on the obtained results, the basic descriptive statistics will be calculated, reported narratively and optionally summarized in a table or figure to support interpretation of studies.

### Data analysis and presentation

The extracted data will be presented in tabular form in a manner that aligns with the objective of this scoping review. A narrative summary will accompany the tabulated results and will describe how the findings relate to the review objective and questions. The main characteristics and results of individual studies will be tabularized as follows:

1. Type of MS used (e.g., MCM-41, SBA-15, KIT-6) with porosity parameters.
2. Name of loaded drug.
3. Applied strategy for improving osseointegration (type and method).
4. Methods and markers used for the evaluation of outcomes.
5. Reported outcomes for osteoinductive, osteoconductive, and proangiogenic properties.

To address which cell lines and *in vivo* or *ex vivo* animal models are most commonly used in studies evaluating osseointegration, we will present the distribution of each cell line, *in vivo/ex vivo* animal model (rows) across osteoconduction, osteoinduction, and angiogenesis research categories (columns) in separate tables. For each cell line, *in vivo* or *ex vivo* animal model we will report both the number of studies and the corresponding percentage. Analogous tables will be prepared for cross-tabulation of methods and markers that are used for the evaluation of osteoconduction, osteoinduction, and angiogenesis. To analyze the drugs used in the included studies, we will (1) calculate the percentage of studies reporting confirmation of drug loading into the MS, (2) categorize drugs into groups (e.g., antimicrobial agents, chemotherapeutics, bisphosphonates), and (3) list the proposed clinical indications for each group of drugs along with the number of studies and the corresponding percentage.

Moreover, we will construct a visual evidence map with MS modifications as rows and outcome domains as columns; within each cell, the shape of markers (triangles, circles, squares) will represent the type of MS (two most common and merged other), color will indicate study design (green for *in vivo*, yellow for *ex vivo*, red for *in vitro* studies), and marker size will reflect the number of studies identified for each combination. Finally, the identified trends across tables and the evidence map will be narratively reviewed.

The analysis of the specific MS bone drug delivery system modification impact on osseointegration will proceed in a structured sequence. Firstly, included studies will be grouped according to the type of MS bone drug delivery system modification and subsequently by the class of loaded drug to determine whether the observed bone-related effect is driven by the intrinsic bioactivity of the drug or by enhancements introduced through modification. This evaluation will only be feasible when appropriate experimental controls are provided (drug-loaded MS system with vs without modifier). In cases where a clear conclusion regarding the relative contribution of the material modification versus the loaded agent cannot be drawn, this will be explicitly acknowledged. The consistency of osseointegration enhancement of the given MS bone drug delivery system modification across various drug types will prevent the overinterpretation of osseointegration properties resulting from highly osteoinductive agents loaded onto MS.

### Ethical declaration

This scoping review protocol and subsequent scoping review do not involve any studies with human participants or animals conducted by authors. Therefore, ethical approval is not required.

## Discussion

The treatment of bone tissue diseases is focused on the local delivery of drugs directly to the affected area, as this is significantly safer and more effective than the general administration of drugs. In this field, mesoporous silica has played an important role for many years, serving as an ideal drug carrier. However, it does not possess sufficient ability to regenerate damaged bone tissue. Therefore, various methods of modifying native mesoporous silica in powder form are applied to ensure the desired bifunctionality and optimal applicability of this drug delivery system. It seems that, so far, there is a lack of systematic gathering of information regarding the performed engineering strategies and their effectiveness at the preclinical stage, leading to many unnecessary attempts repeated by researchers.

Based on the data extracted using this protocol, the scoping review will collect and summarize the engineering strategies applied to drug carriers based on mesoporous silica to improve their bone regenerative effectiveness. Regenerative effectiveness will be divided into the individual processes which are essential elements of successful bone tissue regeneration.

The extracted data will help determine which modifications have been most frequently identified as effective in improving osteoconduction, osteoinduction, or angiogenesis. These results will provide valuable information on the directions for the development of new drug delivery systems based on mesoporous silica, indicating which areas of research should be continued and which should be abandoned. Additionally, the review will allow for the identification of current trends in the utilization of mesoporous silica in more complex systems. Beyond the primary research question, the review will also outline the methods and models used in preclinical studies to evaluate osseointegration, as well as how the findings of these studies are reported.

This scoping review will offer practical guidelines for scientists, engineers, and physicians designing novel bioactive materials for bone tissue regeneration that can also function as carriers for therapeutic agents, enabling them to avoid redundant experiments and concentrate on the most promising approaches. Identification of the most effective engineering strategies will enable and accelerate the development of more advanced regenerative and therapeutic systems.

## Supporting information

S1 Checklist Preferred Reporting Items for Systematic reviews and Meta-Analyses extension for Scoping Reviews Checklist (an adapted version for scoping review protocol). (PDF)

S2 Checklist PRISMA-P (Preferred Reporting Items for Systematic review and Meta-Analysis Protocols) 2015 checklist: recommended items to address in a systematic review protocol* (an adapted version tailored to the specific requirements of the scoping review). (PDF)

S1 Appendix The complete search strategy for PubMed. A data extraction tool developed by the co-authors is available at the following link: https://forms.gle/CHYNyndGDCG9fBeE9.
(DOCX)

## References

[1] McNaught AD, Wilkinson A, editors. The IUPAC Compendium of Chemical Terminology. 2019.

[2] Gisbert-Garzarán M, Manzano M, Vallet-Regí M. Mesoporous Silica Nanoparticles for the Treatment of Complex Bone Diseases: Bone Cancer, Bone Infection and Osteoporosis. Pharmaceutics. 2020;12(1):83. doi: 10.3390/pharmaceutics12010083 31968690 PMC7022913

[3] Leclerc M, Gauvin R. Ordered mesoporous silica: synthesis and applications. In: Functional Materials. 2014. p. 61–100.

[4] Kortesuo P, Ahola M, Karlsson S, Kangasniemi I, Yli-Urpo A, Kiesvaara J. Silica xerogel as an implantable carrier for controlled drug delivery--evaluation of drug distribution and tissue effects after implantation. Biomaterials. 2000;21(2):193–8. doi: 10.1016/s0142-9612(99)00148-9 10632401

[5] Arora M, Arora E. The promise of silicon: bone regeneration and increased bone density. J Arthrosc Jt Surg. 2017;4(3):103–5.

[6] Skwira A, Szewczyk A, Barros J, Laranjeira M, Monteiro FJ, Sądej R, et al. Biocompatible antibiotic-loaded mesoporous silica/bioglass/collagen-based scaffolds as bone drug delivery systems. Int J Pharm. 2023;645. 10.1016/j.ijpharm.2023.123408 37703959

[7] Tamanna T, Landersdorfer CB, Ng HJ, Bulitta JB, Wood P, Yu A. Prolonged and continuous antibacterial and anti-biofilm activities of thin films embedded with gentamicin-loaded mesoporous silica nanoparticles. Appl Nanosci. 2018;8(6):1471–82.

[8] Memar MY, Yekani M, Farajnia S, Ghadiri Moghaddam F, Nabizadeh E, Sharifi S, et al. Antibacterial and biofilm-inhibitory effects of vancomycin-loaded mesoporous silica nanoparticles on methicillin-resistant staphylococcus aureus and gram-negative bacteria. Arch Microbiol. 2023;205(4):109. doi: 10.1007/s00203-023-03447-6 36884153

[9] Szewczyk A, Skwira A, Konopacka A, Sądej R, Walker G, Prokopowicz M. Mesoporous silica pellets as bifunctional bone drug delivery system for cefazolin. Int J Pharm. 2020;588:119718. doi: 10.1016/j.ijpharm.2020.119718 32750441

[10] Balas F, Manzano M, Horcajada P, Vallet-Regí M. Confinement and controlled release of bisphosphonates on ordered mesoporous silica-based materials. J Am Chem Soc. 2006;128(25):8116–7. doi: 10.1021/ja062286z 16787058

[11] Wu C, Fan W, Chang J. Functional mesoporous bioactive glass nanospheres: synthesis, high loading efficiency, controllable delivery of doxorubicin and inhibitory effect on bone cancer cells. J Mater Chem B. 2013;1(21):2710–8. doi: 10.1039/c3tb20275e 32260976

[12] Prokopowicz M, Żeglinski J, Szewczyk A, Skwira A, Walker G. Surface-Activated Fibre-Like SBA-15 as Drug Carriers for Bone Diseases. AAPS PharmSciTech. 2018;20(1):17. doi: 10.1208/s12249-018-1243-5 30574669

[13] Tenland E, Pochert A, Krishnan N, Umashankar Rao K, Kalsum S, Braun K, et al. Effective delivery of the anti-mycobacterial peptide NZX in mesoporous silica nanoparticles. PLoS One. 2019;14(2):e0212858. doi: 10.1371/journal.pone.0212858 30807612 PMC6391042

[14] Orth M, Kruse NJ, Braun BJ, Scheuer C, Holstein JH, Khalil A, et al. BMP-2-coated mineral coated microparticles improve bone repair in atrophic non-unions. Eur Cell Mater. 2017;33:1–12. doi: 10.22203/eCM.v033a01 28054333

[15] Marangoni AC, Campos CES, Capitelli W, Scriboni AB. Research of the osseointegration and saucerization process in bone regeneration for dental implants: a concise systematic review. MedNEXT J Med Heal Sci. 2022;3(3). doi: 10.54448/mdnt22304

[16] Shahrezaie M, Zamanian A, Sahranavard M, Shahrezaee MH. A critical review on the 3D bioprinting in large bone defects regeneration. Bioprinting. 2024;37:e00327. doi: 10.1016/j.bprint.2023.e00327

[17] Albrektsson T, Johansson C. Osteoinduction, osteoconduction and osseointegration. Eur Spine J. 2001;10(Suppl 2):S96-101. doi: 10.1007/s005860100282 11716023 PMC3611551

[18] Grosso A, Burger MG, Lunger A, Schaefer DJ, Banfi A, Di Maggio N. It Takes Two to Tango: Coupling of Angiogenesis and Osteogenesis for Bone Regeneration. Front Bioeng Biotechnol. 2017;5:68. doi: 10.3389/fbioe.2017.00068 29164110 PMC5675838

[19] Burger MG, Grosso A, Briquez PS, Born GME, Lunger A, Schrenk F, et al. Robust coupling of angiogenesis and osteogenesis by VEGF-decorated matrices for bone regeneration. Acta Biomater. 2022;149:111–25. doi: 10.1016/j.actbio.2022.07.014 35835287

[20] Tabbaa SM, Horton CO, Jeray KJ, Burg KJL. Role of vascularity for successful bone formation and repair. Crit Rev Biomed Eng. 2014;42(3–4):319–48. doi: 10.1615/critrevbiomedeng.2014011662 25597242

[21] Florencio-Silva R, Sasso GRdS, Sasso-Cerri E, Simões MJ, Cerri PS. Biology of Bone Tissue: Structure, Function, and Factors That Influence Bone Cells. Biomed Res Int. 2015;2015:421746. doi: 10.1155/2015/421746 26247020 PMC4515490

[22] Gao C, Peng S, Feng P, Shuai C. Bone biomaterials and interactions with stem cells. Bone Res. 2017;5:17059. doi: 10.1038/boneres.2017.59 29285402 PMC5738879

[23] Shen M, Wang L, Feng L, Gao Y, Li S, Wu Y, et al. bFGF-Loaded Mesoporous Silica Nanoparticles Promote Bone Regeneration Through the Wnt/β-Catenin Signalling Pathway. Int J Nanomedicine. 2022;17:2593–608. doi: 10.2147/IJN.S366926 35698561 PMC9188412

[24] Li C, Jiang C, Deng Y, Li T, Li N, Peng M, et al. RhBMP-2 loaded 3D-printed mesoporous silica/calcium phosphate cement porous scaffolds with enhanced vascularization and osteogenesis properties. Sci Rep. 2017;7:41331. doi: 10.1038/srep41331 28128363 PMC5269721

[25] Lozano D, Manzano M, Doadrio JC, Salinas AJ, Vallet-Regí M, Gómez-Barrena E, et al. Osteostatin-loaded bioceramics stimulate osteoblastic growth and differentiation. Acta Biomater. 2010;6(3):797–803. doi: 10.1016/j.actbio.2009.08.033 19716446

[26] Ma J, Lin H, Li X, Bian C, Xiang D, Han X, et al. Hierarchical porous bioactive glasses/PLGA-magnetic SBA-15 for dual-drug release. Mater Sci Eng C Mater Biol Appl. 2014;39:21–8. doi: 10.1016/j.msec.2014.01.060 24863192

[27] Shi M, Xia L, Chen Z, Lv F, Zhu H, Wei F, et al. Europium-doped mesoporous silica nanosphere as an immune-modulating osteogenesis/angiogenesis agent. Biomaterials. 2017;144:176–87. doi: 10.1016/j.biomaterials.2017.08.027 28837959

[28] Kuang Z, Dai G, Wan R, Zhang D, Zhao C, Chen C, et al. Osteogenic and antibacterial dual functions of a novel levofloxacin loaded mesoporous silica microspheres/nano-hydroxyapatite/polyurethane composite scaffold. Genes Dis. 2019;8(2):193–202. doi: 10.1016/j.gendis.2019.09.014 33997166 PMC8099691

[29] Yi M, Nie Y, Zhang C, Shen B. Application of mesoporous silica nanoparticle-chitosan-loaded BMP-2 in the repair of bone defect in chronic osteomyelitis. J Immunol Res. 2022;2022:450196. 10.1155/2022/4450196 PMC9357812 35958879

[30] Liu X, Hu H, Ma J, Wang B. Mineralized cellulose nanofibers reinforced bioactive hydrogel remodels the osteogenic and angiogenic microenvironment for enhancing bone regeneration. Carbohydr Polym. 2025;357:123480. doi: 10.1016/j.carbpol.2025.123480 40159001

[31] Peters MD, Godfrey C, McInerney P, Munn Z, Tricco AC, Khalil H. Scoping reviews. In: JBI Manual for Evidence Synthesis [Internet]. JBI; 2024. Available from: https://jbi-global-wiki.refined.site/space/MANUAL/355862497/10.+Scoping+reviews

[32] Tricco AC, Lillie E, Zarin W, O’Brien KK, Colquhoun H, Levac D, et al. PRISMA Extension for Scoping Reviews (PRISMA-ScR): Checklist and Explanation. Ann Intern Med. 2018;169(7):467–73. doi: 10.7326/M18-0850 30178033

[33] Ought. Elicit [Internet]. Available from: https://elicit.org

[34] Peters MDJ, Marnie C, Tricco AC, Pollock D, Munn Z, Alexander L, et al. Updated methodological guidance for the conduct of scoping reviews. JBI Evid Synth. 2020;18(10):2119–26. doi: 10.11124/JBIES-20-00167 33038124

