# Supplementary material for: Current strategies for improving osseointegration of mesoporous silica drug delivery systems: A scoping review protocol
Source: PLoS One. 2026 Feb 4;21(2):e0338462. doi: 10.1371/journal.pone.0338462 (PMC12871979; doi:10.1371/journal.pone.0338462)
Supplement: S1 Appendix — A data extraction tool developed by the co-authors is available at the following link: https://forms.gle/CHYNyndGDCG9fBeE9. (DOCX) [file pone.0338462.s003.docx]

| # | Description of the search | PubMed | # records  (8.06.2025) |
| --- | --- | --- | --- |
| 1 | broad search of biocompatible coated materials that involve silicon dioxide | ("Coated Materials, Biocompatible"[nm] OR "Coated Materials, Biocompatible"[Mesh]) AND "Silicon Dioxide"[Mesh] | 670 |
| 2 | broad search of drug treatment of bone diseases that involve silicon dioxide | ("Bone Diseases/drug therapy"[Mesh] AND "Silicon Dioxide"[Mesh]) | 80 |
| 3 | porous silica or Si-containing materials | "porous Si"[tiab:~2] OR "porous Sio2"[tiab:~2] OR "porous SiO(2)"[tiab:~2] OR "porous silica"[tiab:~2] OR "porous biosilica"[tiab:~2] | 2853 |
| 4 | mesoporous silica/Si-containing materials | "mesoporous silica"[tiab:~2] OR "mesoporus silica"[tiab:~2] OR "mesoporous silicas"[tiab:~2] OR "mesoporous Si"[tiab:~2] OR "mesoporous Sio2"[tiab:~2] OR "mesoporous Sio(2)"[tiab:~2] OR "mesoporous biosilica"[tiab:~2] OR "mesoporus biosilica"[tiab:~2] | 11502 |
| 5 | ordered or nonordered silica | "silica ordered"[tiab:~2] OR "Si ordered"[tiab:~2] OR "Sio2 ordered"[tiab:~2] OR "Sio(2) ordered"[tiab:~2] OR "silica nonordered"[tiab:~2] OR "Si nonordered"[tiab:~2] OR "Sio2 nonordered"[tiab:~2] OR "Sio(2) nonordered"[tiab:~2] | 910 |
| 6 | mesostructure silica/Si-containing materials | "mesostructure silica"[tiab:~2] OR "mesostructure silica"[tiab:~2] OR "mesostructure silicas"[tiab:~2] OR "mesostructure Si"[tiab:~2] OR "mesostructure Sio2"[tiab:~2] OR "mesostructure Sio(2)"[tiab:~2] OR "mesostructures silica"[tiab:~2] OR "mesostructures silica"[tiab:~2] OR "mesostructures silicas"[tiab:~2] OR "mesostructures Si"[tiab:~2] OR "mesostructures Sio2"[tiab:~2] OR "mesostructures Sio(2)"[tiab:~2] OR "mesostructured silica"[tiab:~2] OR "mesostructured silica"[tiab:~2] OR "mesostructured silicas"[tiab:~2] OR "mesostructured Si"[tiab:~2] OR "mesostructured Sio2"[tiab:~2] OR "mesostructured Sio(2)"[tiab:~2] | 302 |
| 7 | silica or Si-containing particles | "silica particles"[tiab:~2] OR "Si particles"[tiab:~2] OR "Sio2 particles"[tiab:~2] OR "Sio(2) particles"[tiab:~2] OR "biosilica particles"[tiab:~2] | 6746 |
| 8 | silica or Si-containing nanoparticles | "silica nanoparticles"[tiab:~2] OR "Si nanoparticles"[tiab:~2] OR "Sio2 nanoparticles"[tiab:~2] OR "Sio(2) nanoparticles"[tiab:~2] OR "biosilica nanoparticles"[tiab:~2] | 14693 |
| 9 | silica or Si-containing nanospheres | "silica nanospheres"[tiab:~2] OR "silica nanosphere"[tiab:~2] OR "Si nanospheres"[tiab:~2] OR "Si nanosphere"[tiab:~2] OR "Sio2 nanospheres"[tiab:~2] OR "Sio(2) nanospheres"[tiab:~2] OR "Sio2 nanosphere"[tiab:~2] OR "Sio(2) nanosphere"[tiab:~2] OR "biosilica nanospheres"[tiab:~2] OR "biosilica nanosphere"[tiab:~2] | 1148 |
| 10 | silica or Si-containing pellets | "silica pellets"[tiab:~2] OR "Si pellets"[tiab:~2] OR "Sio2 pellets"[tiab:~2] OR "Sio(2) pellets"[tiab:~2] OR "biosilica pellets"[tiab:~2] | 31 |
| 11 | silica or Si-containing scaffolds | "silica scaffold"[tiab:~3] OR "silica scaffolds"[tiab:~3] OR "Si scaffold"[tiab:~2] OR "Si scaffolds"[tiab:~2] OR "SiO2 scaffold"[tiab:~2] OR "SiO2 scaffolds"[tiab:~2] OR "SiO(2) scaffold"[tiab:~2] OR "SiO(2) scaffolds"[tiab:~2] OR "biosilica scaffold"[tiab:~3] OR "biosilica scaffolds"[tiab:~3] | 324 |
| 12 | silica or Si-containing composites | "silica composite"[tiab:~3] OR "silica composites"[tiab:~3] OR "Si composite"[tiab:~2] OR "Si composites"[tiab:~2] OR "SiO2 composite"[tiab:~2] OR "SiO2 composites"[tiab:~2] OR "SiO(2) composite"[tiab:~2] OR "SiO(2) composites"[tiab:~2] OR "biosilica composite"[tiab:~3] OR "biosilica composites"[tiab:~3] | 3316 |
| 13 | silica or Si-containing xerogels | "silica xerogels"[tiab:~2] OR "silica xerogel"[tiab:~2] OR "Si xerogels"[tiab:~2] OR "Sio2 xerogels"[tiab:~2] OR "Sio(2) xerogels"[tiab:~2] OR "biosilica xerogels"[tiab:~2] OR "biosilica xerogel"[tiab:~2] | 250 |
| 14 | silica or Si-containing aerogels | "silica aerogels"[tiab:~2] OR "silica aerogel"[tiab:~2] OR "Si aerogels"[tiab:~2] OR "Sio2 aerogels"[tiab:~2] OR "biosilica aerogels"[tiab:~2] OR "biosilica aerogel"[tiab:~2] | 727 |
| 15 | example of materials of interest: SBA family | "SBA-15"[Supplementary Concept] OR "SBA-15"[Title/Abstract:~1] OR "SBA15"[All Fields] OR "SBA-16"[Supplementary Concept] OR "SBA-16"[Title/Abstract:~1] OR "SBA16"[All Fields] | 2279 |
| 16 | example of materials of interest: MCM family | "MCM-41"[Supplementary Concept] OR "MCM41"[All fields] OR "MCM-41"[tiab:~1] OR "MCM48"[All fields] OR "MCM-48"[tiab:~1] OR "MCM50"[All fields] OR "MCM-50"[tiab:~1] OR "MCM mesoporous"[tiab:~5] | 2104 |
| 17 | example of materials of interest: KIT family | "KIT6"[All Fields] OR "KIT 6"[All Fields] OR "KIT5"[All Fields] OR "KIT 5"[All Fields] OR "KIT mesoporous"[Title/Abstract:~5] | 309 |
| 18 | example of materials of interest: HMM family | "HMM1"[All Fields] OR "HMM-1"[All Fields] OR "HMM2"[All Fields] OR "HMM-2"[All Fields] OR "HMM mesoporous"[Title/Abstract:~5] | 19 |
| 19 | example of materials of interest: HMS family | "HMS mesoporous"[tiab:~5] | 107 |
| 20 | example of materials of interest: COK family | "COK12"[All Fields] OR "COK-12"[All Fields] OR "COK-12"[tiab:~1] OR "COK19"[All Fields] OR "COK-19"[All Fields] OR "COK-19"[tiab:~1] OR "COK mesoporous"[Title/Abstract:~5] | 8 |
| 21 | example of materials of interest: FSM family | "FSM16"[All fields] OR "FSM-16"[tiab:~1] OR "FSM mesoporous"[tiab:~5] | 43 |
| 22 | example of materials of interest: MSU family | "MSU mesoporous"[tiab:~5] | 27 |
| 23 | brand names of materials of interest | "AEROPERL 300"[tiab:~2] OR "Aeroperl300"[All fields] OR "AEROPERL silica"[tiab:~10] OR "AEROPERL mesoporous"[tiab:~10] OR "Syloid XDP"[Title/Abstract:~3] OR "XDP3050"[All Fields] OR "Syloid FP"[Title/Abstract:~3] OR "Syloid silica"[Title/Abstract:~10] OR "Syloid mesoporous"[Title/Abstract:~10] OR Silsol[All fields] OR "Parteck SLC"[tiab:~5] OR "Sylysia"[All fields] | 123 |
| 24 | silica or Si-based materials | "silica-based"[tiab:~2] OR "sillica-based"[tiab:~2] OR "Si-based"[tiab:~2] OR "SiO2-based"[tiab:~2] OR "SiO(2)-based"[tiab:~2] OR "biosilica-based"[tiab:~2] | 9403 |
| 25 | Functionalized or modified silica materials | "Functionalized silica"[Title/Abstract:~3] OR "Functionalised silica"[Title/Abstract:~3] OR "Functionalized SiO2"[Title/Abstract:~3] OR "Functionalised SiO2"[Title/Abstract:~3] OR "functionalized sio 2"[Title/Abstract:~3] OR "functionalised sio 2"[Title/Abstract:~3] OR "Bifunctionalized silica"[Title/Abstract:~3] OR "Bifunctionalised silica"[Title/Abstract:~3] OR "Bifunctionalized SiO2"[Title/Abstract:~3] OR "Bifunctionalised SiO2"[Title/Abstract:~3] OR "bifunctionalized sio 2"[Title/Abstract:~3] OR "bifunctionalised sio 2"[Title/Abstract:~3] OR "Modified silica"[Title/Abstract:~3] OR "Modified SiO2"[Title/Abstract:~3] OR "modified sio 2"[Title/Abstract:~3] OR "Grafted silica"[Title/Abstract:~3] OR "Grafted SiO2"[Title/Abstract:~3] OR "grafted sio 2"[Title/Abstract:~3] OR "Functionalized biosilica"[Title/Abstract:~3] OR "Functionalised biosilica"[Title/Abstract:~3] OR "Bifunctionalized biosilica"[Title/Abstract:~3] OR "Bifunctionalised biosilica"[Title/Abstract:~3] OR "Modified biosilica"[Title/Abstract:~3] OR "Grafted biosilica"[Title/Abstract:~3] | 8173 |
| 26 | Merge 1-25 | #1 OR #2 OR #3 OR #4 OR #5 OR #6 OR #7 OR #8 OR #9 OR #10 OR #11 OR #12 OR #13 OR #14 OR #15 OR #16 OR #17 OR #18 OR #19 OR #20 OR #21 OR #22 OR #23 OR #24 OR #25 | 45393 |
| 27 | Osteoconduction | "Bone Regeneration"[Mesh] OR "Bone regeneration"[All fields] OR "Bone regeneration"[tiab:~2] OR "osteoconduction"[All Fields] OR "osteoconductive"[All Fields] OR "osteoconductivity"[All Fields] OR "osteoconduc*"[tiab] OR "osseoconduction"[All Fields] OR "osseoconductive"[All Fields] OR "osseoconductivity"[All Fields] OR "osseoconduc*"[Title/Abstract] OR "Osseointegration"[Mesh] OR Osseointegrat*[tiab] OR "Endosseous Healing"[tiab:~2] | 56009 |
| 28 | Osteoinduction | "Bone Development"[Mesh] OR "Osteogenesis"[Mesh] OR "osteoinduction"[All Fields] OR "osteoinductive"[All Fields] OR "osteoinducting"[All Fields] OR "osteoinductivity"[All Fields] OR "osseoinduction"[All Fields] OR "osseoinductive"[All Fields] OR "osseoinductivity"[All Fields] OR "osteoinduct*"[Title/Abstract] OR "osseoinduct*"[Title/Abstract] | 81672 |
| 29 | Bone cells | osteoblast*[tiab] OR osteoclast*[tiab] OR osteocyte*[tiab] OR "preosteoblast*"[tiab] OR "pre-osteoblast"[tiab] OR "preosteocyt*"[tiab] OR "preosteoclast*"[tiab] OR "osteoprogenitor*"[tiab] | 94980 |
| 30 | Bone cell lines | "hFOB"[tiab] OR "hFOB1.19"[tiab] OR "MC3T3-E1"[tiab] OR "SaOS-2"[tiab] OR "MG-63"[tiab] OR "U2OS"[tiab] OR "U2 OS"[tiab] OR "143B"[tiab] OR "UMR-106"[tiab] OR "ROS 17/2.8"[tiab] OR "HOS cells"[tiab:~2] OR "HOS osteosarcoma"[tiab:~2] OR "KUSA-A1"[tiab] OR "2T3 cells"[tiab:~2] OR "SV-HFO"[tiab] | 19356 |
| 31 | Proteins of interest: SIBLINGs | "small integrin-binding ligand N-linked glycoprotein"[All fields] OR "Small Integrin-Binding Ligand N-linked Glycoproteins"[All fields] | 111 |
| 32 | Proteins of interest: decorin or biglycan | "Decorin"[Mesh] OR "Biglycan"[Mesh] OR "decorin*"[tiab] OR "biglycan*"[tiab] OR "bone proteoglycan"[tiab:~2] OR "bone proteoglycans"[tiab:~2] | 4385 |
| 33 | Proteins of interest: osteopontin | "Osteopontin"[Mesh] OR "osteopontin*"[tiab] OR "Bone Sialoprotein" OR "OPN"[tiab] OR "BSP"[tiab] OR "SPP1"[tiab] | 21862 |
| 34 | Proteins of interest: Osteocalcin | "Osteocalcin"[Mesh] OR "osteocalcin*"[tiab] OR "Bone GLA Protein"[tiab] OR "BGLAP"[tiab] OR "OCN"[tiab] | 24410 |
| 35 | Proteins of interest: Osteonectin | "Osteonectin"[Mesh] OR "osteonectin*"[tiab] OR "SPARC Glycoprotein"[tiab:~2] OR "BM-40"[tiab] | 3045 |
| 36 | Proteins of interest: osteoprotegerin | "Osteoprotegerin"[Mesh] OR "Osteoprotegerin"[All fields] OR "Osteoclastogenesis Inhibitory Factor"[tiab] OR "OPG"[tiab] OR "TNFRSF11B"[tiab] | 11764 |
| 37 | Proteins of interest: Bone Sialoprotein 2 | "Integrin-Binding Sialoprotein"[Mesh] OR "Integrin-Binding Sialoprotein"[tiab] OR "bone sialoprotein"[tiab:~2] OR "IBSP"[tiab] | 2988 |
| 38 | Proteins of interest: alkaline phosphatase | "Alkaline Phosphatase"[Mesh] OR "alkaline phosphatase"[tiab] OR "ALP"[tiab] | 116211 |
| 39 | Proteins of interest: type 1 collagen (including 1A1) | "Collagen Type I"[Mesh] OR "Collagen Type I, alpha 1 Chain"[Mesh] OR "COL1A1"[tiab] OR "COL1"[tiab] OR "Type 1 Collagen"[tiab:~3] OR "Type I Collagen"[tiab:~3] | 48106 |
| 40 | Proteins of interest: bone morphogenetic proteins (BMP-2, BMP-4, BMP7) | "Bone Morphogenetic Proteins"[Mesh] OR "Bone Morphogenetic Protein"[tiab:~3] OR "Osteogenic Protein"[tiab] OR "BMP2"[tiab] OR "BMP4"[tiab] OR "BMP7"[tiab] | 35779 |
| 41 | Proteins of interest: RANK-L | "RANK Ligand"[Mesh] OR "RANK ligand"[tiab] OR "RANK-L"[tiab] OR "Receptor-activator of NF-kappaB ligand"[tiab] OR "TNFSF11"[tiab] OR "OPGL"[tiab] OR "TNF-related activation-induced cytokine"[tiab] | 10284 |
| 42 | Proteins of interest: Sclerostin | "SOST protein, human"[Supplementary Concept] OR sclerostin[tiab] OR "SOST"[tiab] | 3450 |
| 43 | Proteins of interest: Integrin α1β1 | "Integrin alpha1beta1"[Mesh] OR "alpha1beta1 integrin"[tiab:~4] OR "alpha1beta1 integrins"[tiab:~4] OR "alpha1 beta1 integrin"[tiab:~4] OR "alpha1 beta1 integrins"[tiab:~4] | 644 |
| 44 | Proteins of interest: Integrin α2β1 | "Integrin alpha2beta1"[Mesh] OR "alpha2beta1 integrin"[tiab:~4] OR "alpha2beta1 integrins"[tiab:~4] OR "alpha2 beta1 integrin"[tiab:~4] OR "alpha2 beta1 integrins"[tiab:~4] | 1552 |
| 45 | Proteins of interest: integrin α5β1 | "Integrin alpha5beta1"[Mesh] OR "alpha5beta1 integrin"[tiab:~4] OR "alpha5beta1 integrins"[tiab:~4] OR "alpha5 beta1 integrin"[tiab:~4] OR "alpha5 beta1 integrins"[tiab:~4] | 2815 |
| 46 | Proteins of interest: integrin αvβ3 | "Integrin alphaVbeta3"[Mesh] OR "alphaVbeta3 integrin"[tiab:~4] OR "alphaVbeta3 integrins"[tiab:~4] OR "alphaV beta3 integrin"[tiab:~4] OR "alphaV beta3 integrins"[tiab:~4] | 5313 |
| 47 | Proteins of interest: integrin αvβ5 | "integrin alphaVbeta5"[Supplementary Concept] OR "alphaVbeta5 integrin"[tiab:~4] OR "alphaVbeta5 integrins"[tiab:~4] OR "alphaV beta5 integrin"[tiab:~4] OR "alphaV beta5 integrins"[tiab:~4] | 1082 |
| 48 | Transcription factors | "RUNX2"[tiab] OR "RUNT-RELATED TRANSCRIPTION FACTOR 2"[tiab] OR "Core Binding Factor Alpha 1 Subunit"[Mesh] OR "Sp7 Transcription Factor"[Mesh] OR "Transcription factor Sp7"[tiab] OR "Osterix"[tiab] | 14128 |
| 49 | Staining: cellular mineralization | "Alizarin Red S" [Supplementary Concept] OR "Alizarin Red"[All fields] OR "Alizarin Red"[tiab:~2] OR "von Kossa"[tiab] OR "Arsenazo III"[MeSH Terms] OR "Arsenazo III"[All Fields] OR "Arsenazo III"[Title/Abstract:~2] | 9313 |
| 50 | Angiogenesis, vascularization oraz tubulogenesis | "Angiogenesis"[MeSH] OR "angiogen*"[Title/Abstract] OR "proangio*"[Title/Abstract] OR "vasculari*"[Title/Abstract] OR "provasculari*"[Title/Abstract] OR "revascular*"[tiab] OR "tubulogenes*"[tiab] | 309063 |
| 51 | angiogenesis: protein of interest (VEGF) | "Vascular Endothelial Growth Factors"[Mesh] OR "Vascular Endothelial Growth Factor"[All fields] OR "VEGF*"[tiab] | 131452 |
| 52 | angiogenesis: protein of interest (angiopoetin-1 and -2, angiopoietin-like protein 4) | "Angiopoietins"[Mesh] OR "Angiopoietin-1"[Mesh] OR "Angiopoietin-2"[Mesh] OR "Angiopoietin-Like Protein 4"[Mesh] OR "Angiopoietin"[tiab] OR "ANGPT1"[tiab] OR "ANGPT2"[tiab] OR "Angiopoietin Like Protein 4"[tiab:~1] OR "ANGPTL4"[tiab] | 9434 |
| 53 | angiogenesis: protein of interest (placental growth factor) | "Placenta Growth Factor"[Mesh] OR "Placenta Growth Factor"[tiab:~2] OR "Placenta Growth Factors"[tiab:~2] OR "Placental Growth Factor"[tiab:~2] OR "Placental Growth Factors"[tiab:~2] OR "PlGF"[All fields] | 5073 |
| 54 | angiogenesis: TGF-beta1 | "Transforming Growth Factor beta1"[Mesh] OR "Transforming Growth Factor-beta1"[All fields] OR "TGF-beta-1"[All fields] OR "TGF-beta1"[All fields] | 50171 |
| 55 | angiogenesis: protein of interest (FGF) | "fibroblast growth factor 1"[MeSH Terms] OR "fibroblast growth factor 2"[MeSH Terms] OR "fibroblast growth factor 9"[MeSH Terms] OR "Fibroblast growth factor 18"[Supplementary Concept] OR "FGF-1"[Title/Abstract] OR "FGF1"[Title/Abstract] OR "fibroblast growth factor 1"[Title/Abstract] OR "FGF-2"[Title/Abstract] OR "FGF2"[Title/Abstract] OR "bFGF"[Title/Abstract] OR "fibroblast growth factor 2"[Title/Abstract] OR "FGF-9"[Title/Abstract] OR "FGF9"[Title/Abstract] OR "fibroblast growth factor 9"[Title/Abstract] OR "FGF-18"[Title/Abstract] OR "FGF18"[Title/Abstract] OR "Fibroblast growth factor 18"[Title/Abstract] | 29405 |
| 56 | angiogenesis: protein of interest (PDGF) | "Platelet-Derived Growth Factor"[Mesh] OR "Platelet-Derived Growth Factor"[tiab] OR "PDGF"[tiab] | 31275 |
| 57 | Vascular endothelium cell lines | "Human Umbilical Vein Endothelial Cells"[Mesh] OR "Human Umbilical Vein Endothelial Cell"[tiab] OR "HUVEC"[tiab] OR "Human Dermal Microvascular Endothelial Cells"[tiab] OR "HMEC-1"[tiab] OR "HDMEC"[tiab] OR "Porcine Iliac Artery Endothelial Cells"[tiab] OR "PIEC"[tiab] OR "EA.hy926"[tiab] OR "HIAEC"[tiab] OR "hPAEC cells"[tiab:~2] OR "LEII cells"[tiab:~3] OR "LEII endothelial"[tiab:~3] OR "MLEC cells"[tiab:~3] OR "MLEC endothelial"[tiab:~3] OR HCAEC[tiab] OR HCAECs[tiab] OR "hCMEC/D3"[tiab] OR "HPMEC"[tiab] OR "HREC cells"[tiab:~3] OR "HREC endothelial"[tiab:~3] OR "TY10 cells"[tiab:~3] OR "TY10 endothelial"[tiab:~3] OR "RF/6A"[tiab] OR "H5V"[tiab] OR "bEnd.3"[tiab] OR "Bone marrow endothelial cells"[All Fields] OR "BMEC endothelial"[tiab:~3] OR "BMECs endothelial"[tiab:~3] | 35093 |
| 58 | angiogenesis: tests | "angiogenesis assay"[tiab:~6] OR "angiogenesis assays"[tiab:~6] OR "chorioallantoic membrane"[tiab] OR "CAM assay"[All fields] OR "CD31"[tiab] OR "tube forming assay"[tiab:~3] OR "tube forming assays"[tiab:~3] OR "Fibrin Gel assay"[tiab:~3] OR (("wound healing assay"[tiab] OR "wound closure assay"[tiab] OR "Trans-well cell migration assay"[tiab] OR "Transwell cell migration assay"[tiab] OR "Trans-well migration assay"[tiab] OR "Transwell cell migration assay"[tiab]) AND endothelial[tiab]) | 24817 |
| 59 | Merge 27-58 | #27 OR #28 OR #29 OR #30 OR #31 OR #32 OR #33 OR #34 OR #35 OR #36 OR #37 OR #38 OR #39 OR #40 OR #41 OR #42 OR #43 OR #44 OR #45 OR #46 OR #47 OR #48 OR #49 OR #50 OR #51 OR #52 OR #53 OR #54 OR #55 OR #56 OR #57 OR #58 | 867014 |
| 60 | Filter to exclude studies not related to drug delivery systems (to exclude studies examining silica particles without any drug) | "Drug Delivery Systems"[Mesh] OR "Drug Implants"[Mesh] OR "Drug Compounding"[Mesh] OR "deliver*"[tiab] OR "carrier*"[tiab] OR "vehicle*"[tiab] OR "drug*"[tiab] OR "active substance"[tiab:~2] OR "active agent"[tiab:~2] OR "active ingredient"[tiab:~2] OR "active compound"[tiab:~2] OR "load*"[tiab] OR "adsorb*"[tiab] OR "absorb*"[tiab] OR "contain*"[tiab] OR "releas*"[tiab] | 6405260 |
| 61 | Final strategy | #26 AND #59 AND #60 AND (2015/1/1:3000/12/12[pdat]) | 716 |
